# Beyond BMI: Rethinking Obesity Metrics and Cardiovascular Risk in the Era of Precision Medicine

**DOI:** 10.3390/diagnostics15233025

**Published:** 2025-11-27

**Authors:** Maria-Daniela Tanasescu, Andrei-Mihnea Rosu, Alexandru Minca, Andreea-Liana Rosu, Maria-Mihaela Grigorie, Delia Timofte, Dorin Ionescu

**Affiliations:** 1 Department of Semiology-Emergency University Hospital, Carol Davila University of Medicine and Pharmacy, 022328 Bucharest, Romania; maria.tanasescu@umfcd.ro (M.-D.T.);; 2 Department of Cardiology, Prof. Dr. Agrippa Ionescu Emergency Hospital, 077015 Balotesti, Romania; 3 Department of Clinical Pharmacology, BBraun, 013714 Bucharest, Romania; 4 Department of Dentistry, Discipline of Endodontics, Faculty of Dentistry, Carol Davila University of Medicine and Pharmacy, 020021 Bucharest, Romania; 5 Department of Dialysis, Bucharest Emergency University Hospital, 050098 Bucharest, Romania

**Keywords:** BMI, obesity phenotypes, cardiometabolic risk, visceral adiposity, precision medicine, body composition, metabolic health, cardiovascular disease

## Abstract

Obesity remains a dominant risk factor for cardiovascular disease, yet its classification continues to rely heavily on body mass index (BMI)—a metric that fails to capture individual variability in fat distribution, metabolic health, and cardiometabolic risk. This narrative review analyzes 35 articles published between 2018 and 2025 to explore the limitations of BMI and outlines emerging strategies for obesity redefinition through a precision medicine lens. Drawing from recent advances in imaging, metabolomics, and genomic profiling, we highlight alternative metrics such as visceral adipose tissue (VAT), epicardial adipose tissue (EAT), waist-to-hip ratio (WHR), and multi-omic phenotyping that provide superior predictive value for cardiovascular outcomes. The review synthesizes data on metabolically healthy and unhealthy phenotypes, emphasizes the pathophysiological role of EAT in heart failure and arrhythmogenesis, and discusses the cardioprotective effects of pharmacologic agents such as glucagon-like peptide-1 (GLP-1) receptor agonists. Clinical implications include improved risk stratification, earlier disease detection, and individualized therapeutic targeting. Despite current barriers to widespread implementation—such as imaging cost, access to omics, and lack of guideline integration—this paradigm shift holds promise for refining cardiovascular prevention strategies. Redefining obesity using biologically informed, phenotype-based models is indispensable for aligning clinical practice with the complexities of modern cardiometabolic disease.

## 1. Introduction

Since its introduction in the early 1970s, Body Mass Index (BMI) has served as the cornerstone for defining and classifying obesity in clinical and public health contexts. Originating from the work of Ancel Keys and colleagues, BMI offered a convenient, population-level proxy for body fat content, enabling cross-national comparisons and epidemiological surveillance with minimal resources [1]. The metric was rapidly adopted by global health authorities, most notably the World Health Organization (WHO), which formally endorsed BMI-based cut-offs for overweight (≥25 kg/m^2^) and obesity (≥30 kg/m^2^) in its 2000 technical report—cementing its status as the global standard [2]. Owing to its simplicity and reproducibility, BMI has been widely used to monitor trends, guide interventions, and assess risk stratification across diverse populations. However, as pointed out in subsequent critiques, its utility at the individual level remains limited, as it fails to distinguish between lean and fat mass or account for ethnic and sex-based differences in body composition [3]. These limitations are particularly pertinent in the context of modern precision medicine, which demands more nuanced and physiologically relevant markers for cardiometabolic risk assessment.

Despite its widespread adoption, BMI has increasingly come under scrutiny for its inability to reflect individual variability in cardiometabolic health. Individuals with identical BMI values may exhibit markedly different physiological profiles—ranging from insulin sensitivity and lipid levels to inflammatory markers and visceral fat accumulation—resulting in distinct levels of cardiovascular risk [4]. This heterogeneity has led to the recognition of divergent obesity phenotypes, including the so-called metabolically healthy obese (MHO) and metabolically unhealthy normal weight (MUNW) individuals. While the former present with a high BMI but favorable metabolic profiles, the latter may have normal BMI values despite harboring significant metabolic dysfunction. Such phenotypes illustrate the disconnect between BMI and actual health status, emphasizing the metric’s limited discriminative capacity in both clinical and preventive cardiology [5].

Emerging evidence from large-scale population studies supports the use of metabolic profiling and multi-omic analysis to uncover risk patterns that BMI alone cannot detect. These approaches have revealed that some individuals within the same BMI category may exhibit drastically different lipidomic and proteomic signatures, correlating with divergent cardiometabolic trajectories. Moreover, the reliance on BMI as a universal metric fails to account for population-specific factors, including ethnic differences in fat distribution, muscle mass, and inflammation—all of which modulate cardiovascular risk independently of total body weight [6,7]. Clinical guidelines have increasingly emphasized the importance of evaluating fat distribution—particularly visceral and ectopic fat—as these are more closely linked to cardiovascular outcomes than BMI-defined obesity alone [8]. As such, a paradigm shift is underway toward more granular, phenotype-based models of obesity classification that better align with the goals of precision medicine.

Advances in precision medicine have highlighted the biological complexity hidden within BMI-defined obesity. Polygenic risk scores (PRSs), derived from genome-wide association studies, have shown that genetic predisposition to obesity involves distinct pathways: central nervous system genes often influence appetite regulation and overall adiposity, while adipose tissue-related genes shape fat distribution and metabolic outcomes [9].

In parallel, metabolomic profiling reveals extensive biochemical variation among individuals with similar BMI. Up to one-third of the human plasma metabolome is altered in obesity, yet the direction and intensity of these changes differ across individuals, enabling the identification of metabolically distinct subtypes [10]. Tools such as metabolomic BMI (mBMI) can differentiate between metabolically unhealthy normal-weight individuals and metabolically healthy obese individuals with higher clinical accuracy than anthropometrics alone. While PRSs offer limited predictive power in isolation, integrating genetic and metabolomic data enhances cardiometabolic risk stratification [11].

The emergence of advanced imaging technologies has revealed that total body weight alone is a poor predictor of cardiometabolic risk. Instead, regional fat distribution—particularly the accumulation of visceral adipose tissue (VAT)—has been shown to be more strongly associated with adverse cardiovascular outcomes than subcutaneous fat or BMI-defined obesity. VAT is a metabolically active depot, characterized by a higher density of proinflammatory immune cells and increased insulin resistance, contributing to an endocrine environment conducive to atherogenesis, hypertension, and insulin resistance [12].

Cross-sectional modalities such as MRI, CT, and DEXA scans allow for precise quantification of VAT and other ectopic fat depots, offering superior risk stratification in both obese and normal-weight individuals [13]. Notably, VAT drains into the portal circulation, directly affecting hepatic metabolism and promoting dyslipidemia, glucose intolerance, and systemic inflammation. This direct link accentuates its pathophysiological relevance in the progression of cardiovascular disease (CVD). Moreover, imaging studies have identified that VAT volume correlates more robustly with adverse outcomes—such as left ventricular hypertrophy, arterial stiffness, and coronary calcification—than BMI or waist circumference. Recent advances in artificial intelligence have enabled the development of deep learning models that combine chest radiographs with clinical variables to estimate VAT with high accuracy, potentially democratizing access to precision cardiometabolic risk profiling. Such approaches may outperform traditional anthropometric methods and provide actionable insight into patient-specific fat phenotypes [14]. As precision medicine becomes increasingly central to cardiology, VAT quantification is poised to become a routine component of cardiovascular risk assessment.

In light of the limitations of BMI and the growing body of evidence supporting a biologically grounded classification of obesity, this review aims to re-examine how adiposity is defined and measured—particularly in the context of cardiovascular risk. We explore emerging alternatives to BMI, including multi-omic profiling, polygenic risk scoring, and imaging-based fat distribution metrics, with a focus on their relevance to cardiology and precision medicine. Special attention is given to the paradoxes of metabolically healthy obesity and metabolically unhealthy normal weight, and how these phenotypes challenge traditional assumptions about weight-related cardiovascular risk. By synthesizing recent findings from genetic, metabolic, and imaging research, we advocate for a redefinition of obesity that better aligns with pathophysiological mechanisms and individualized risk stratification. The review concludes by highlighting future directions and the clinical implications of adopting a precision-based approach to obesity in cardiovascular care.

## 2. Materials and Methods

This narrative review was conducted to synthesize current perspectives on the limitations of BMI and the emerging role of alternative metrics in cardiometabolic risk assessment, particularly within the context of precision medicine. To identify relevant literature, a targeted search was carried out using PubMed, Scopus, and Web of Science databases. Additional sources were identified through manual screening of the reference lists of selected articles, ensuring that recent advances and influential studies not captured by automated searches were also considered. The search was conducted in September 2025 and covered literature published between January 2018 and September 2025.

The search strategy included terms and Boolean combinations such as “obesity phenotypes,” “BMI limitations,” “visceral adiposity,” “metabolically healthy obesity,” “polygenic risk score,” “metabolomics,” “body composition imaging,” “cardiometabolic risk,” and “precision cardiology.” These terms were chosen to reflect the review’s primary focus on redefining obesity through multi-parametric approaches that integrate imaging, molecular, and genetic data, as well as the clinical implications of obesity-related phenotypes in cardiovascular care.

Two reviewers independently screened titles and abstracts for relevance, followed by full-text assessment of eligible articles. Discrepancies were resolved through discussion and consensus. Although this is a narrative rather than a systematic review, efforts were made to enhance methodological transparency by applying selected elements of systematic review reporting frameworks. Given the narrative nature of this review, a formal PRISMA framework was not applied, and no standardized risk of bias assessment tools were used. Formal quality appraisal tools were not appropriate due to the conceptual and exploratory aims of this synthesis, which integrates heterogeneous evidence types (e.g., omics studies, imaging protocols, and mechanistic models). Instead, emphasis was placed on conceptual integration, recency of evidence, and translational relevance to cardiovascular and precision medicine.

The included studies were synthesized using a narrative, thematic approach. Articles were grouped based on conceptual overlap into four major domains: (1) limitations of BMI in cardiovascular risk stratification; (2) imaging-based assessments of adiposity; (3) omics-driven obesity classification (including genomics and metabolomics); and (4) cardiological implications of phenotype-based definitions. This allowed for integrative comparison of evidence across methods and clinical contexts.

Studies were selected based on their relevance to adult human populations and their contribution to understanding obesity’s role in cardiovascular disease beyond BMI classification. Peer-reviewed original research articles, narrative reviews, and systematic reviews were included, provided they were published in English and aligned with the clinical and conceptual domains of interest. Articles focusing exclusively on pediatric populations, surgical or bariatric interventions, or animal models were excluded, as were case reports, editorials, and non-peer-reviewed materials.

A total of 35 articles were ultimately included in the review. The selected articles were grouped thematically to reflect four overarching areas: the limitations of BMI in cardiovascular risk stratification, imaging-based assessments of adiposity, omics-driven obesity classification, and cardiological implications of phenotype-based definitions.

Because this review follows a narrative design, no formal meta-analysis or quality appraisal tools were applied. Instead, emphasis was placed on conceptual integration, recent evidence, and the translational relevance of findings to clinical cardiology. The review aims to provide a comprehensive, yet focused synthesis of current knowledge, offering a framework for redefining obesity in the era of precision medicine.

**Figure 1:**
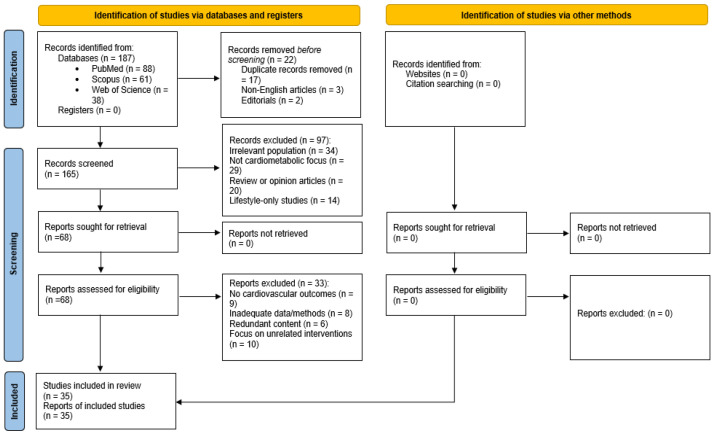
PRISMA 2020 flow diagram detailing the study selection process.

To enhance methodological transparency, a PRISMA 2020 flow diagram has been included (Figure 1), summarizing the identification, screening, eligibility assessment, and inclusion of articles. This diagram is presented to increase transparency of the literature selection process, even though formal systematic procedures such as bias scoring and meta-analysis were not performed.

## 3. Results

### 3.1. Limitations of BMI in Cardiovascular Risk Prediction

Although BMI remains a cornerstone in defining obesity, it is a limited tool for assessing cardiometabolic risk. It does not differentiate between fat and lean mass, nor does it account for fat distribution—both of which are key contributors to the development of CVD. Recent research highlights substantial heterogeneity in obesity-related health outcomes, with some individuals exhibiting high BMI but low cardiometabolic risk. This phenomenon, often described as the “obesity paradox” or “fit-fat paradox,” challenges the assumption that excess body weight, as defined by BMI, uniformly predicts adverse cardiovascular outcomes [15].

One of the clearest examples of this heterogeneity is the MHO phenotype. Individuals classified as MHO meet BMI criteria for obesity yet do not exhibit metabolic disturbances such as insulin resistance, dyslipidemia, or hypertension. Compared to their metabolically unhealthy obese (MUO) counterparts, MHO individuals typically show preserved insulin sensitivity, lower visceral and hepatic fat, and higher cardiorespiratory fitness [16]. However, MHO is not a stable condition. Longitudinal evidence suggests that many individuals initially classified as MHO transition over time to a metabolically unhealthy state, with a concomitant increase in CVD risk [16,17].

Nevertheless, elevated cardiometabolic risk is not limited to individuals with obesity. Those with normal BMI but poor metabolic profiles—referred to as MUNW—may face comparable or even greater cardiovascular risk than MHO individuals. This phenotype is often characterized by increased visceral fat, impaired insulin sensitivity, and elevated proinflammatory markers despite a “normal” BMI [17]. Thus, BMI alone cannot reliably differentiate between cardiometabolically benign and high-risk phenotypes.

A summary of the clinical and metabolic features associated with MHO, MUO, and MUNW is presented in Table 1.

The limitations of BMI become particularly evident when considering its inability to differentiate between lean and fat mass or capture central adiposity. This is critical in cardiovascular risk prediction, where visceral fat, rather than total body weight, is a stronger determinant of adverse outcomes [18]. Individuals with similar BMI values may differ substantially in their visceral fat content, which directly influences insulin resistance, lipid metabolism, and systemic inflammation.

A large-scale meta-analysis evaluating the discriminatory capacity of anthropometric indices for CVD found that both waist circumference (WC) and waist-to-hip ratio (WHR) outperformed BMI in predicting cardiovascular events. The pooled area under the curve (AUC) for BMI was 0.66, whereas WC and WHR had AUCs of 0.69 and 0.71, respectively—particularly among women—indicating superior predictive capacity for central adiposity markers [19].

BMI’s application across diverse populations is further complicated by ethnic and sex-based differences in body composition. For instance, individuals of Asian descent may develop obesity-related complications at lower BMI thresholds, while those with greater muscle mass, such as athletes, may be misclassified as obese. In older adults, age-related muscle loss may lead to underestimation of adiposity using BMI alone, thereby obscuring risk [18].

Beyond these technical limitations, framing obesity solely through BMI has broader clinical implications. BMI neither measures fat distribution nor accurately estimates body fat percentage and fails to capture the metabolic, genetic, and physiological diversity of obesity [20]. Consequently, BMI may overlook MUNW, while misclassifying some MHO individuals as high-risk despite a favorable metabolic profile.

In response to these shortcomings, updated diagnostic frameworks have emerged. The Commission on Clinical Obesity proposes categorizing obesity into “preclinical” and “clinical” stages based on the presence of functional impairments and organ-specific dysfunction. In this model, BMI serves only as an initial screening tool. Diagnostic confirmation requires further assessment through direct adiposity measurements—such as dual-energy X-ray absorptiometry (DEXA) or bioelectrical impedance analysis (BIA)—or waist-based anthropometric indices [21]. This shift prioritizes physiological relevance over rigid weight-based thresholds.

**Figure 2:**
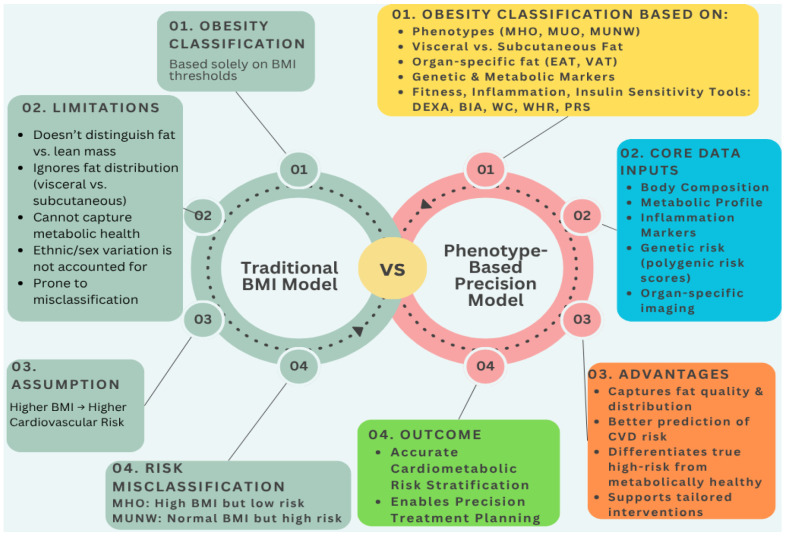
Conceptual Comparison of Obesity Classification Models.

In order to illustrate the conceptual shift from conventional obesity assessment to a precision-based approach, Figure 2 compares the traditional BMI model with a multi-dimensional, phenotype-driven framework for cardiometabolic risk evaluation.

### 3.2. Alternative Metrics of Obesity in Precision Medicine

WHR has emerged as a superior anthropometric marker of cardiometabolic risk, surpassing BMI and waist circumference in predictive power. Large cohort studies, including the Northern Finland Birth Cohort and UK Biobank, demonstrated that WHR more effectively captures risk related to β-cell dysfunction, insulin resistance, and type 2 diabetes, even when controlling for BMI and lifestyle factors [22,23,24]. Furthermore, WHR correlates with metabolomic markers of inflammation, dyslipidemia, and hepatic steatosis, reinforcing its relevance in early detection and risk stratification [25,26].

It has to be noted that WHR offers enhanced discriminatory ability across sexes due to its sensitivity to sex-specific adipose distribution patterns: gluteofemoral dominance in women and abdominal accumulation in men [27]. In female cohorts with cardiovascular disease, WHR-based phenotyping identified distinct metabolic profiles not reflected in BMI, underlining its added clinical value [28].

VAT, distinguishable from subcutaneous adipose tissue (SAT), has an important role in metabolic dysregulation, contributing to inflammation, insulin resistance, and atherogenesis [29,30]. While WHR serves as a practical surrogate for central adiposity in large populations, imaging modalities such as CT and MRI offer direct quantification of VAT and SAT, enabling more precise phenotyping.

CT, due to its high resolution and Hounsfield unit-based tissue contrast, remains the reference standard for VAT quantification but is limited by radiation exposure. MRI provides radiation-free imaging with excellent anatomical detail, though its utility is constrained by lower signal contrast and segmentation complexity. DEXA, while useful for whole-body composition estimates, lacks the spatial resolution to differentiate VAT from SAT. These technical distinctions should guide their use in research versus clinical settings.

Automated and semi-automated segmentation algorithms, particularly deep learning-based models such as convolutional neural networks (CNNs), have shown high accuracy in VAT and SAT segmentation. These approaches offer scalable solutions that reduce radiologist workload and improve reproducibility across sites. For example, a 2D U-Net applied to water–fat MRI demonstrated excellent performance across multiple centers, with Dice coefficients above 0.97 for VAT and SAT and quantification errors below 3% [31]. In another study, a Cycle-GAN and U-Net pipeline enabled VAT segmentation from unannotated MRI data by generating synthetic CT images, validated through radiologist ratings [32]. CT-based phenotyping using AI also showed that VAT distribution correlates more strongly with type 2 diabetes risk than BMI, even among individuals with normal weight [33]. Comparison of VAT segmentation tools demonstrated high inter-model agreement (Cohen’s κ = 0.856), supporting the use of validated pipelines in clinical research workflows [34].

A detailed comparison of these AI-based adipose tissue segmentation studies is presented in Table 2.

DEXA has been increasingly recognized as a more precise alternative to BMI in the assessment of obesity and associated cardiometabolic risk. Evidence indicates that BMI significantly underestimates obesity prevalence when compared to DEXA-derived body fat percentage (BF%), particularly in populations with distinct ethnic and physiological profiles [35]. It has been demonstrated that the BMI threshold of 30 kg/m^2^ fails to accurately reflect adiposity in some groups, and a lower BMI cut-off may be more appropriate when DEXA is used as the reference standard. DEXA has also been validated for its ability to reliably quantify VAT. Studies have confirmed that VAT measured via DEXA correlates strongly with cardiometabolic biomarkers and provides reproducible estimates across diverse body sizes [36]. Moreover, DEXA has proven useful in identifying phenotypes such as NWO, characterized by normal BMI but excess VAT, which would otherwise go undetected through traditional anthropometric indices [37]. Comparative studies between DEXA and CT have demonstrated that while CT remains the gold standard for regional adiposity, DEXA provides clinically acceptable estimates of VAT and lean mass with lower cost, radiation exposure, and greater accessibility [38].

To contextualize the comparative utility of various obesity assessment tools in precision medicine, Table 3 summarizes the general strengths, limitations, and clinical applications of WHR, DEXA, CT, and MRI as phenotyping methods.

While numerous studies support the prognostic value of EAT volume, important methodological limitations remain. Imaging protocols vary considerably across studies—CT, MRI, and echocardiography each introduce unique biases, limiting comparability. Cut-off values for “high-risk” EAT or VAT differ widely and are not yet standardized or validated across diverse populations. Moreover, echocardiographic assessment of EAT is highly operator-dependent and lacks consistent measurement protocols, leading to limited reproducibility. Additionally, the predictive performance of VAT and EAT may vary by age, sex, ethnicity, and comorbid conditions, which are often underrepresented in existing cohorts. These factors highlight the need for greater harmonization in imaging methodology and more inclusive validation efforts.

### 3.3. Integrating Genetics and Multi-Omics

The integration of multi-omics approaches has provided critical insights into the complex molecular architecture of obesity. While genome-wide association studies (GWAS) have identified numerous loci associated with BMI, the functional interpretation and clinical translation of these findings remain limited. Multi-omics strategies combining genetic, transcriptomic, epigenomic, and proteomic data offer a more comprehensive framework for identifying molecular determinants of obesity and refining cardiometabolic risk stratification.

Recent advances in multi-omics research are redefining our understanding of the molecular architecture underlying obesity. Rather than treating obesity as a homogenous condition defined solely by BMI, integrative genomic approaches now allow for the dissection of tissue-specific and pathway-specific mechanisms. For instance, a large genome-wide multi-omics meta-analysis by Tang et al. [39], which combined GWAS, transcriptome wide (TWAS), and epigenome wide (EWAS) data from over 350,000 individuals, identified 195 genes significantly associated with BMI. Among these, 21 were linked to adipose tissue networks and 53 to brain-specific networks, with 11 genes overlapping both. This dual enrichment pinpoints the role of the brain–adipose axis in regulating energy balance and body weight.

Immune and inflammatory signaling within adipose tissue have also emerged as key contributors to obesity pathogenesis. Li et al. [40] employed transcriptomics and single-cell RNA sequencing (scRNA-seq) to identify TREM2 and CXCR4 as central regulators of macrophage-driven inflammation and fibrosis in obese adipose tissue. Their work demonstrated that TREM2 is selectively expressed in lipid-associated macrophages and was reproducibly validated across both murine and human datasets, implicating it in obesity-induced immune dysfunction.

Beyond identifying single-gene drivers, multi-omics profiling is now uncovering molecular phenotypes that diverge from traditional anthropometric indicators. As highlighted by Hu and Jia [41], individuals with similar BMI may exhibit markedly different molecular signatures, particularly in lipid metabolism, inflammatory tone, and insulin responsiveness. This observation has been further supported by Aleksandrova et al. [42], who argued that omics-based classification strategies can reveal metabolically unhealthy individuals who are misclassified as low-risk based on BMI alone. Their findings also accentuate the limitations of BMI as a universal risk metric and highlight the need for individualized, biology-driven assessments.

The translational potential of these insights is perhaps most evident in obesity-related comorbidities such as non-alcoholic fatty liver disease (NAFLD). Using a targeted multi-omics approach, Diels et al. [43] identified paraoxonase-1 (PON1) as a key determinant of hepatic steatosis severity. Reduced expression and activity of PON1 were linked to oxidative stress, lipid dysregulation, and liver inflammation. As their results imply, integrating proteomic and imaging data improved early detection of NAFLD and suggested that PON1 may serve as both a biomarker and therapeutic target in obesity-associated liver disease.

Epigenetic regulation also appears to play a dynamic role in obesity risk and progression. In a multi-omics analysis, Zhang et al. [44] showed that histone methylation patterns in genes such as PPARG, LEPTIN, and TNF are closely associated with adiposity, adipocyte hypertrophy, and inflammation. These modifications were found to be tissue- and context-specific, suggesting their utility in disease staging and metabolic risk stratification.

At a systems level, obesity is increasingly being conceptualized not as a single phenotype but as a spectrum defined by distinct biological axes. Odoemelam et al. [45] proposed a four-axis model—encompassing general adiposity, hepatic fat, systemic inflammation, and muscle composition—derived from integrative imaging and multi-omics data. Each axis reflects unique molecular signatures that align with specific cardiometabolic risk trajectories, offering a more granular alternative to BMI-based classification.

All of these insights are not limited to risk stratification but are beginning to inform personalized intervention strategies. Woldemariam et al. [46] demonstrated that integrating genomic, transcriptomic, metabolomic, and microbiomic profiles can stratify individuals into discrete molecular endotypes. These endotypes, characterized by features such as mitochondrial dysfunction, branched-chain amino acid metabolism, and chronic inflammation, are now being explored as the basis for tailoring lifestyle and pharmacologic interventions to the patient’s unique molecular profile.

All the studies mentioned illustrate the promise of multi-omics approaches in redefining obesity as a heterogeneous, biologically stratified condition—moving beyond weight-centric paradigms toward a framework grounded in metabolic function and molecular pathophysiology.

### 3.4. Epicardial Adipose Tissue: A Cardiometabolic Biomarker in Obesity Redefinition

Recent findings suggest that the cardiovascular consequences of obesity extend beyond what is measurable by BMI, supporting a shift toward more nuanced definitions based on metabolic function and ectopic fat distribution. Adipose tissue, particularly when deposited viscerally and around the heart as epicardial adipose tissue, plays an active role in cardiovascular remodeling. In obesity, hypertrophied adipocytes become metabolically dysregulated, secreting elevated levels of inflammatory cytokines including TNF-α, IL-6, and MCP-1, which contribute to systemic insulin resistance, endothelial dysfunction, and myocardial fibrosis. These changes underpin the development of HFpEF, which is increasingly linked to metabolic obesity, even in the absence of overt hyperglycemia or elevated BMI. The role of lipotoxicity has also gained attention, with excessive accumulation of lipid intermediates such as ceramides and diacylglycerols within cardiomyocytes contributing to cellular stress, apoptosis, and impaired contractility. These cardiometabolic effects are further exacerbated in individuals with metabolically unhealthy phenotypes, regardless of body size. Imaging-based studies confirm that increased EAT volume is independently associated with left ventricular hypertrophy, subclinical diastolic dysfunction, and coronary atherosclerosis, and may better predict cardiovascular risk than BMI or waist circumference alone [47]. As a surrogate of visceral adiposity and metabolic risk, EAT is also modifiable, and emerging pharmacologic strategies target this depot directly. Glucagon-like peptide-1 receptor agonists (GLP-1RAs), widely used for obesity and type 2 diabetes, have demonstrated pleiotropic benefits that include reductions in systemic inflammation, improvement of myocardial energetics, and attenuation of adverse structural remodeling. In patients with HFpEF, GLP-1RAs were associated with improvements in symptoms, exercise tolerance, and selected biomarkers of cardiac stress. These cardioprotective effects appeared to be at least partially independent of weight loss or glycemic control, and were more pronounced in individuals with concurrent obesity and metabolic dysfunction [48]. Recent evidence from the STEP-HFpEF trial has solidified their role in obesity-related cardiac dysfunction. In this randomized controlled study involving 529 patients with HFpEF and obesity, once-weekly semaglutide significantly improved symptoms, physical function, and biomarkers of cardiac stress—independent of glycemic status or weight loss alone. Patients receiving semaglutide experienced greater reductions in NT-proBNP (−21%), CRP levels, and improved KCCQ-CSS scores (+16.6 points) compared to placebo. These cardiometabolic improvements were more pronounced in those with visceral adiposity, supporting the hypothesis that therapies targeting ectopic fat depots such as epicardial adipose tissue may offer pleiotropic benefits in obesity-related HFpEF [49]. Nevertheless, variability in study designs, heterogeneity in patient populations, and insufficient stratification by heart failure phenotypes limit definitive conclusions. The cardiometabolic benefits of GLP-1RAs are supported by outcome trials showing a reduction in major adverse cardiovascular events (MACE), particularly stroke and cardiovascular mortality, with modest effects on heart failure hospitalizations. While these therapies may not yet be routinely applied for HF management, their favorable profile in obese individuals at risk of cardiovascular events stresses the need to redefine obesity through a cardiocentric and phenotype-specific lens [50]. This includes re-evaluating risk in those with metabolically healthy obesity or metabolically unhealthy normal weight, who may harbor unrecognized subclinical myocardial changes despite falling outside traditional BMI-based risk thresholds.

The role of EAT in cardiac structure and function is being redefined from a passive fat deposit to an active endocrine organ influencing myocardial remodeling and electrophysiological stability. Recent echocardiographic evidence in middle-aged adults shows that incremental increases in EAT thickness are associated with graded changes in left ventricular wall thickness, left atrial dilation, and impaired diastolic function, independent of BMI, waist circumference, or blood pressure. This suggests a continuous rather than threshold-based impact of EAT on cardiac morphology and function [51]. It is important to note that these alterations occur even in younger populations, indicating that subclinical remodeling related to visceral fat may begin decades before overt cardiovascular disease becomes manifest. EAT appears to exert its effects via direct paracrine mechanisms due to its unique anatomic proximity to the myocardium, lacking any fascial separation. In elderly hypertensive patients, EAT thickness measured via transthoracic echocardiography was found to predict the 2-year incidence of atrial fibrillation (AF) with high reproducibility. Each 1 mm increase in EAT was associated with a 62% greater risk of incident AF, and a cut-off value of 6.5 mm effectively stratified high-risk individuals. This relationship remained significant even after adjusting for traditional risk factors such as BMI, blood pressure, diabetes, and atrial size, emphasizing the independent predictive value of EAT [52]. Furthermore, adding EAT to clinical risk models significantly improved discrimination, calibration, and reclassification metrics. The presence of elevated EAT not only contributes to atrial remodeling but also aligns with elevated inflammatory mediators and diastolic filling abnormalities—early indicators of heart failure with HFpEF. Complementing this, editorial perspectives stress the need for longitudinal studies and multi-omic profiling to further elucidate the biological mechanisms linking EAT to myocardial disease, while also highlighting ethnic variability and the modifying effects of pharmacologic agents such as GLP-1 receptor agonists [53]. Taken together, these insights support a redefinition of obesity that prioritizes fat distribution and metabolic behavior over crude anthropometric indices. Integrating EAT measurement into routine echocardiographic evaluations may represent a low-cost, high-yield strategy for early cardiovascular risk detection and precision-guided intervention in obese and metabolically vulnerable populations.

To complement the narrative findings, Table 4 and Table 5 provide an integrated summary of the key clinical correlates and imaging-based metrics of EAT in the context of obesity-related cardiac remodeling, as reported in recent studies [47,48,49,50,51,52,53,54].

## 4. Discussion

### 4.1. Summary of Key Findings

This review highlights the limitations of BMI as a universal marker of cardiometabolic risk and underscores the need for a paradigm shift toward more biologically grounded, phenotype-driven approaches. Emerging evidence consistently shows that metrics such as VAT, EAT, WHR, and multi-omic biomarkers offer significantly greater predictive power for cardiovascular outcomes than BMI alone [15,16,17,19,47]. Furthermore, the delineation of obesity phenotypes—such as MHO and MUNW—demonstrates the inadequacy of BMI in capturing true metabolic risk [4,16]. Integrating advanced imaging, PRS, and metabolomic profiling enables more accurate stratification and paves the way for precision-guided interventions.

This narrative review expands upon the recent quantitative syntheses that support the superior predictive value of WHR and VAT over BMI. For example, a meta-analysis [56] of over 700,000 participants found that WHR is significantly associated with increased myocardial infarction risk, especially in women and Asian populations (OR = 1.98, 95% CI: 1.75–2.24). Similarly, a classical meta-regression [57] demonstrated that each 0.01 unit increase in WHR is associated with a 5% higher risk of cardiovascular events, independent of BMI. A more recent population-based imaging study [58] showed that VAT, but not abdominal subcutaneous adipose tissue, was independently predictive of coronary heart disease (HR per 1-SD VAT = 1.15, 95% CI: 1.09–1.22), and this association persisted even after adjustment for waist circumference.

While the reviewed studies generally support the superiority of VAT, EAT, and omics-based metrics over BMI, the evidence is not uniformly consistent. Some cohorts report weaker associations in elderly or ethnically diverse populations, raising concerns about generalizability. In addition, while certain studies emphasize the predictive strength of EAT volume via CT, others using echocardiographic thickness report more modest associations, reflecting methodological variability. These inconsistencies call for standardization across imaging modalities and validation across demographic groups. Despite these differences, the emerging consensus supports a shift away from BMI-centric frameworks toward a more nuanced, phenotype-based understanding of cardiometabolic risk.

### 4.2. Clinical and Translational Implications

The clinical implications of these findings are substantial. Cardiologists and primary care providers must recognize that two patients with similar BMI may have different risk trajectories due to differences in fat distribution, inflammation, and metabolic flexibility [18,47]. Imaging-based assessments of VAT and EAT provide actionable, non-invasive biomarkers that correlate more strongly with subclinical cardiac remodeling, atherosclerosis, and heart failure progression than traditional anthropometric indices [12,50]. Importantly, pharmacologic agents like GLP-1 receptor agonists appear to influence these fat depots independently of weight loss, offering new therapeutic angles that transcend BMI-defined treatment pathways [48,49]. A recent meta-analysis [59] of 29 studies including 19,709 patients, confirmed that higher EAT measurements, quantified either by CT-derived volume or echocardiographic thickness, are independently associated with an increased risk of MACE. In adjusted analyses, each 1-SD increase in EAT volume was associated with a 42% higher risk of MACE (aHR = 1.42; 95% CI, 1.22–1.65), and individuals in the highest EAT volume category had more than double the risk (aHR = 2.64; 95% CI, 1.23–5.67). CT-derived EAT volume was more predictive (aHR = 1.74) than EAT thickness by echocardiography (aHR = 1.20). Secondary outcomes also showed that elevated EAT was associated with significantly increased odds of cardiac death (OR = 2.53), myocardial infarction (OR = 2.63), coronary revascularization (OR = 2.99), and atrial fibrillation (aOR = 4.04). These important results reinforce the clinical utility of EAT quantification as a non-invasive biomarker for cardiovascular risk stratification.

These findings also have potential implications for public health frameworks and clinical policy. As evidence accumulates on the limitations of BMI in capturing metabolic risk, international bodies such as the WHO and cardiology societies like the ESC and ACC may consider revising obesity definitions and risk stratification tools to incorporate phenotype-specific markers, including WHR, VAT quantification, and omics-based classifiers. It is important to note that regulatory interest in obesity-related metrics is growing. For instance, recent FDA label expansions and clinical guidance have acknowledged the cardioprotective effects of GLP-1 receptor agonists in patients with HFpEF, independent of weight loss. This shows a broader shift in regulatory thinking—from weight-centric to cardiometabolic-centric endpoints—which supports integrating more precise, mechanistically grounded obesity metrics into clinical guidelines.

### 4.3. Limitations of Current Evidence

Despite these promising developments, several limitations persist in the existing literature. Many omics-based studies lack long-term follow-up, limiting their predictive value for hard cardiovascular outcomes. Ethnic, sex-based, and age-related differences in fat distribution are often underrepresented or underpowered in cohort studies, raising concerns about generalizability [7,28]. Additionally, while deep learning–based tools for VAT and EAT quantification show high accuracy in research settings, clinical implementation remains constrained by cost, infrastructure, and expertise [31,32]. The integration of multi-modal data into electronic health records is another major bottleneck in translating precision obesity metrics into everyday practice.

### 4.4. Challenges in Clinical Adoption

Adopting a phenotype-based model in clinical cardiology faces structural and educational hurdles. Clinicians remain trained in BMI-centric paradigms, and practice guidelines have yet to fully endorse alternative metrics such as VAT, WHR, or PRS. In lower-resource settings, access to MRI or DEXA may be limited, and omics data may not be readily available or interpretable. To mitigate these challenges, simplified clinical tools that approximate VAT or EAT using accessible surrogates—like advanced echocardiography or AI-enhanced radiographs—could serve as transitional steps toward full precision implementation [14,52].

### 4.5. Future Research and Policy Directions

Future studies should aim to standardize the measurement and interpretation of alternative obesity metrics across populations and care settings. Longitudinal, multi-ethnic cohort studies are needed to evaluate how combinations of imaging, omics, and anthropometric data predict major adverse cardiovascular events over time. Additionally, the development of integrated risk scores that include EAT thickness, metabolomic BMI, and inflammatory markers could enhance risk stratification, particularly for MUNW individuals who are often overlooked in traditional frameworks [10,45]. At a policy level, redefining obesity to include functional and distributional measures of adiposity could prompt a shift in diagnostic thresholds and treatment eligibility criteria.

## 5. Conclusions

The continued reliance on BMI as a primary marker of obesity-related cardiovascular risk is increasingly misaligned with advances in precision medicine. This review emphasizes the inadequacy of BMI in capturing individual differences in fat distribution, metabolic health, and genetic susceptibility. Phenotypes such as MHO and MUNW exemplify the disconnect between body size and cardiovascular risk, accentuating the urgent need to redefine obesity beyond weight-based cutoffs.

Alternative metrics—WHR, VAT, EAT, and multi-omic biomarkers—offer more physiologically relevant, predictive, and individualized assessments. Imaging modalities and molecular profiling tools have demonstrated the ability to stratify cardiometabolic risk more accurately, enabling early detection of subclinical disease and informing personalized interventions.

As cardiology increasingly embraces precision approaches, the clinical paradigm must shift from a “one-size-fits-all” BMI model to an integrated framework that considers adipose tissue distribution, function, and molecular signatures. Moving forward, redefining obesity through a cardiocentric, multi-parametric lens will be essential to improving risk prediction, optimizing treatment strategies, and aligning clinical practice with the biological realities of metabolic disease. To bridge the gap between precision research and real-world cardiology, future obesity assessment should integrate VAT and EAT quantification into standard cardiovascular risk evaluations. Similarly, incorporating metabolic profiling—including lipidomics, inflammatory markers, and polygenic risk scores—can enhance individualized risk prediction. Emerging digital health tools and artificial intelligence-based imaging platforms offer scalable solutions for phenotyping adiposity with greater accuracy and lower cost. As these technologies become more accessible, clinicians will be better equipped to implement phenotype-based obesity management strategies that move beyond BMI, enabling earlier intervention and more targeted cardiometabolic care.

## 6. Strengths and Limitations

This review offers a timely and comprehensive synthesis of emerging obesity classification models that transcend traditional BMI-based frameworks. By integrating findings from multi-omics, imaging modalities, and population-level phenotype studies, the review provides a cohesive, translational narrative that bridges metabolic research and cardiovascular practice. It incorporates recent high-impact literature (2018–2025), including genome-wide association studies, advanced imaging analyses, and metabolomic profiling, making the review particularly relevant for clinicians and researchers engaged in precision cardiology.

As a narrative review, this work does not apply formal meta-analytic techniques or systematic bias assessments, which may limit the reproducibility of literature inclusion and synthesis. Furthermore, narrative approaches are susceptible to selection and confirmation biases, and may inadvertently reflect prevailing paradigms in the field. While omics-based metrics hold great potential for refining cardiometabolic risk prediction, they remain limited by methodological and translational challenges. These include small and often homogeneous sample sizes, high inter-study heterogeneity, lack of assay and platform standardization, elevated costs, and underdeveloped clinical pipelines for data interpretation.

The review primarily focuses on adult populations, with limited discussion of pediatric obesity phenotypes or age-specific trajectories of metabolic risk. While it draws on diverse global data sources, representation from low- and middle-income countries remains limited, potentially constraining generalizability. Additionally, rapid technological developments in omics and imaging may render certain conclusions provisional, pending broader clinical validation and standardization.

## Figures and Tables

**Table 1:** Key Characteristics of Obesity Phenotypes: MHO, MUO, and MUNW.

Feature	MHO	MUO	MUNW
BMI	≥30 kg/m^2^	≥30 kg/m^2^	18.5–24.9 kg/m^2^
VAT	Low (≤120 cm^2^) [15]	High (≥160 cm^2^) [15,16]	High (≥130 cm^2^) [17]
Subcutaneous Leg Fat	High (qualitative; protective) [15,17]	Low [15,16]	Low [17]
Hepatic Steatosis	Low (<5% liver fat) [15]	High (>5% liver fat) [15,17]	High (>5% liver fat) [17]
Insulin Sensitivity	Preserved (HOMA-IR < 2.5) [15,17]	Impaired (HOMA-IR > 2.5–3.0) [16,17]	Impaired [17]
Inflammatory Markers	Low (hsCRP < 1.0 mg/L) [15]	Elevated (hsCRP > 3.0 mg/L) [15,16]	Elevated [17]
Blood Pressure	Normal (<130/85 mmHg) [15,16]	Elevated (≥130/85 mmHg) [15,16]	Elevated [17]
Cardiorespiratory Fitness	Higher (VO_2_ max > 30 mL/kg/min) [16]	Lower (VO_2_ max < 25 mL/kg/min) [16]	Variable [17]
Cardiovascular Risk	Moderate (ASCVD risk 10–20%) [15]	High (>20% ASCVD risk or Framingham score) [15,17]	High (>20% risk) [17]
Phenotype Stability	Transient (6–16% remain stable over time) [17]	Persistent [16]	Variable, often unstable [17]

Abbreviations: MHO—Metabolically Healthy Obesity; MUO—Metabolically Unhealthy Obesity; MUNW—Metabolically Unhealthy Normal Weight; BMI—Body Mass Index; VAT—Visceral Adipose Tissue; HOMA-IR—Homeostatic Model Assessment of Insulin Resistance; hsCRP—High-Sensitivity C-Reactive Protein; VO_2_ max—Maximal Oxygen Uptake; ASCVD—Atherosclerotic Cardiovascular Disease.

**Table 2:** Summary of AI-Based Adipose Tissue Segmentation Studies.

Study	Imaging Modality	AI Model	Tissue Type(s)	Performance Metric	Dataset/Population	Key Outcomes
[31] Langer et al., 2019	Water–fat MRI	2D U-Net	VAT, SAT	Dice: VAT 0.970 ± 0.015; SAT 0.987 ± 0.011; Error < 3%	Multi-center (UK Biobank subset)	High accuracy; suitable for large-scale studies
[32] Masoudi et al., 2020	MRI (via Cycle-GAN → sCT)	Cycle-GAN + U-Net	VAT, SAT	Radiologist rating: VAT 3.80/5; SAT 4.54/5	NIH MRI (n = 34), CT (n = 131)	Enables VAT segmentation without MRI annotations
[33] Remedios et al., 2025	CT	Random Forest + UMAP	VAT, SAT, organs	AUC = 0.72–0.74 for T2DM prediction	1728 adults from Vanderbilt	VAT predicts diabetes risk better than BMI
[34] Hou et al., 2024	CT	nnU-Net vs. TotalSegmentator	VAT, SAT, muscle	Cohen’s κ = 0.856 (VAT); Dice: SAT 83.8 vs. 80.8; Muscle 87.6 vs. 83.2	SAROS dataset (n = 900 scans)	Internal tool outperformed public pipeline

VAT = Visceral Adipose Tissue; SAT = Subcutaneous Adipose Tissue; sCT = synthetic CT; UMAP = Uniform Manifold Approximation and Projection; T2DM = Type 2 Diabetes Mellitus.

**Table 3:** Comparison of key obesity phenotyping methods used in precision medicine.

Metric	WHR	DEXA	CT	MRI
Phenotyping focus	Central fat distribution (ratio-based)	Total body fat, lean mass, estimated VAT	Compartment-specific VAT/SAT quantification	High-resolution VAT/SAT mapping
Clinical relevance	Good predictor of metabolic risk, especially when combined with BMI	Differentiates obesity phenotypes; correlates with metabolic markers	Gold standard for VAT; strong association with cardiometabolic outcomes	Provides detailed fat distribution profiles; useful for mechanistic studies
Radiation exposure	None	Low	High	None
Cost and accessibility	Very low; globally scalable	Moderate; widely used in clinical practice	High; limited to research and specialized centers	High; less available in routine care
Segmentation and interpretation	Manual, anthropometric	Automated or manual via device software	High precision; supports AI-driven segmentation	Requires advanced segmentation algorithms; supports deep learning tools
Limitations	Does not assess visceral fat directly; affected by body proportions	Limited resolution for VAT vs. SAT separation	Ionizing radiation; less suited for frequent monitoring	Time-intensive; low contrast between fat and soft tissue
Best use case	Population screening, low-resource settings	Individual risk stratification and fat distribution profiling	Research or clinical validation of adiposity metrics	Exploratory studies or advanced metabolic phenotyping

**Table 4:** Clinical Correlates of EAT.

Clinical Feature	Key Findings	Clinical Implications
EAT and Inflammation [48,50,55]	Secretion of IL-6, TNF-α, MCP-1; low adiponectin	Promotes endothelial dysfunction, CAD, and fibrosis; involved in myocardial remodeling and systemic inflammation in HF
Neurohormonal Activation [47,55]	Activates RAAS and TGF-β pathways	Promotes myocardial fibrosis in HFpEF
Lipotoxicity [47]	EAT releases ceramides, DAGs, ROS	Contributes to arrhythmias and cardiomyocyte apoptosis
Subclinical Cardiac Remodeling [51]	EAT thickness linked to ↑ LV wall, LA dilation, ↓ diastolic velocities	Suggests early cardiac remodeling in midlife
AF Risk Prediction [52]	EAT >6.5 mm = HR 5.1 for incident AF; +1 mm = HR 1.62	Independent predictor of AF in elderly hypertensive patients
Cardiometabolic Disease in Diabetes [50,55]	EAT linked to insulin resistance, endothelial dysfunction; correlates with T2DM in HFpEF	Suggests EAT as a systemic metabolic risk factor; guides comorbidity management in HFpEF
Therapeutic Modifiability [47,49]	Liraglutide and semaglutide reduce EAT; improve symptoms and CRP	Supports EAT as treatment target
Myocardial Ischemia and MACE [54]	OR 1.062 (ischemia), HR 1.040 (MACE) per 10 cm^3^ EAT	Independent prognostic marker in low/intermediate risk
Graded Risk Effect [51]	Echocardiographic risk ↑ across EAT thickness deciles	EAT impact is continuous, not threshold-dependent
Association with CAD and Severity [54]	Higher EAT volume and thickness in CAD and severe CAD	Validates diagnostic and staging utility of EAT

Abbreviations: AF, atrial fibrillation; CAD, coronary artery disease; CRP, C-reactive protein; DAGs, diacylglycerols; EAT, epicardial adipose tissue; HFpEF- heart failure with preserved ejection fraction; HR, hazard ratio; IL-6, interleukin-6; LA, left atrium; LV, left ventricle; MACE, major adverse cardiovascular events; MCP-1, monocyte chemoattractant protein-1; RAAS, renin–angiotensin–aldosterone system; ROS, reactive oxygen species; TGF-β, transforming growth factor-beta; TNF-α, tumor necrosis factor-alpha.

**Table 5:** Imaging Metrics for Epicardial Adipose Tissue (EAT) in Cardiovascular Risk Assessment.

Imaging Modality	Key Findings	Clinical Implications
Echocardiography	EAT thickness predicts incident AF: 1 mm ↑ in EAT = HR 1.62 (cutoff 6.5 mm) [52] EAT thickness associated with LV wall thickness, LA dilation, ↓ e′ velocities in midlife adults [51] EAT often misidentified as pericardial effusion [47] EAT thickness correlates with impaired GLS, independent of BMI [48]	Supports inclusion of EAT in echo protocols Enables early detection of subclinical cardiac remodeling Cutoff of 6.5 mm for AF risk stratification
Computed Tomography (CT)	EAT volume > 125 cm^3^ linked with ↑ coronary calcium score, plaque burden, and subclinical atherosclerosis [48,50]; per 10 cm^3^ increase in EAT, OR for obstructive CAD = 1.055 and MACE = 1.040 [54] Severe CAD has higher EAT volume than mild/moderate (SMD: 0.33; *p* = 0.0007) [53]	CT quantifies EAT precisely for risk stratification May improve prediction of CAD, MACE, and ischemia in low-risk populations
Tissue Doppler Imaging (TDI)	Detects diastolic dysfunction associated with EAT accumulation [47]	Useful for functional cardiac assessment in EAT-related remodeling
SPECT/PET	SPECT imaging affected by fat artifacts; PET provides better accuracy in EAT-laden patients [47]	Imaging protocols should be adjusted in obese patients to account for EAT interference

Abbreviations: AF, atrial fibrillation; CAD, coronary artery disease; CT, computed tomography; EAT, epicardial adipose tissue; GLS, global longitudinal strain; HR, hazard ratio; LA, left atrium; LAVi, left atrial volume index; LV, left ventricle; MACE, major adverse cardiovascular events; PET, positron emission tomography; SMD, standardized mean difference; SPECT, single-photon emission computed tomography; TDI, tissue Doppler imaging.

## Data Availability

No new data were created or analyzed in this study. Data sharing is not applicable to this article.

## Abbreviations

The following abbreviations are used in this manuscript:

AI	Artificial Intelligence
BIA	Bioelectrical Impedance Analysis
BMI	Body Mass Index
CNN	Convolutional Neural Network
CT	Computed Tomography
CVD	Cardiovascular Disease
DEXA	Dual-Energy X-ray Absorptiometry
EAT	Epicardial Adipose Tissue
EF	Ejection Fraction
HDL	High-Density Lipoprotein
HF	Heart Failure
HU	Hounsfield Unit
MACE	Major Adverse Cardiovascular Events
MHO	Metabolically Healthy Obese
Mbmi	Metabolomic Body Mass Index
MRI	Magnetic Resonance Imaging
MUNW	Metabolically Unhealthy Normal Weight
MUO	Metabolically Unhealthy Obese
NAFLD	Non-Alcoholic Fatty Liver Disease
PRS	Polygenic Risk Score
SAT	Subcutaneous Adipose Tissue
TNF	Tumor Necrosis Factor
VAT	Visceral Adipose Tissue
WC	Waist Circumference
WHR	Waist-to-Hip Ratio
2D U-Net	Two-Dimensional U-shaped Convolutional Network
